# Beyond DNA Repair: DNA-PKcs in Tumor Metastasis, Metabolism and Immunity

**DOI:** 10.3390/cancers12113389

**Published:** 2020-11-16

**Authors:** Haitang Yang, Feng Yao, Thomas M. Marti, Ralph A. Schmid, Ren-Wang Peng

**Affiliations:** 1 Department of Thoracic Surgery, Shanghai Chest Hospital, Shanghai Jiao Tong University, Shanghai 200030, China; haitang.yang@dbmr.unibe.ch (H.Y.); yaofeng6796678@126.com (F.Y.); 2 Division of General Thoracic Surgery, Inselspital, Bern University Hospital, CH3008 Bern, Switzerland; thomas.marti@insel.ch; 3 Department of BioMedical Research (DBMR), University of Bern, 3012 Bern, Switzerland

**Keywords:** DNA-dependent protein kinase catalytic subunit, DNA damage response, metastasis, metabolism, immunity, biomarker, targeted therapy, immunotherapy

## Abstract

**Simple Summary:**

DNA-dependent protein kinase catalytic subunit (DNA-PKcs) has recently attracted increasing attention due to the emerging novel functions in cancer. However, several fundamental questions, such as the underlying molecular basis for DNA-PKcs pleiotropy and biomarkers for DNA-PKcs inhibitor sensitivity, remain to be addressed. In this review, we elaborate an updated summary of the most recent progress in DNA-PKcs, with particular emphasis on the mechanisms of DNA-PKcs multifaceted roles in malignancy and potential stratification approaches towards DNA-PKcs-based precision cancer therapy.

**Abstract:**

The DNA-dependent protein kinase catalytic subunit (DNA-PKcs) is a key component of the DNA-PK complex that has a well-characterized function in the non-homologous end-joining repair of DNA double-strand breaks. Since its identification, a large body of evidence has demonstrated that DNA-PKcs is frequently overexpressed in cancer, plays a critical role in tumor development and progression, and is associated with poor prognosis of cancer patients. Intriguingly, recent studies have suggested novel functions beyond the canonical role of DNA-PKcs, which has transformed the paradigm of DNA-PKcs in tumorigenesis and has reinvigorated the interest to target DNA-PKcs for cancer treatment. In this review, we update recent advances in DNA-PKcs, in particular the emerging roles in tumor metastasis, metabolic dysregulation, and immune escape. We further discuss the possible molecular basis that underpins the pleiotropism of DNA-PKcs in cancer. Finally, we outline the biomarkers that may predict the therapeutic response to DNA-PKcs inhibitor therapy. Understanding the functional repertoire of DNA-PKcs will provide mechanistic insights of DNA-PKcs in malignancy and, more importantly, may revolutionize the design and utility of DNA-PKcs-based precision cancer therapy.

## 1. Introduction

The DNA-dependent protein kinase (DNA-PK) is a large holoenzyme composed of the DNA-PK catalytic subunit (DNA-PKcs) and a heterodimer of Ku proteins (Ku70/Ku80) [1]. It was originally identified as part of the SP1 (specificity protein 1) transcription complex and as a regulatory component of transcriptionally poised RNA polymerase II (RNAPII) [2]. Subsequently, it has been studied in detail concerning its function in DNA double-strand break (DSB) repair, especially in the non-homologous end joining (NHEJ) repair pathway [3,4].

Consistent with the role in maintaining genomic instability, a hallmark of cancer, a considerable number of studies have demonstrated the association between dysregulated DNA-PKcs and cancer development [5,6,7,8]. Overexpression of DNA-PKcs is frequent in a variety of cancer types and predicts poor prognosis in patients [9,10,11,12,13,14]. Consequently, DNA-PKcs has been shown to be a potential therapeutic target, especially in combination with radio- and chemotherapy that induce DNA damage [2,15,16].

Intriguingly, recent studies have revealed novel functions of DNA-PKcs in cancer, such as cell cycle progression, tumor metastasis and resistance to therapy, metabolic regulation, and tumor immunity [7,17,18,19,20,21], which transforms the paradigm of DNA-PKcs and expands the utility of DNA-PKcs inhibitors in cancer treatment. Nevertheless, the molecular underpinnings for the pleiotropy of DNA-PKcs remain largely unknown.

In the light of an increasing spectrum of DNA-PKcs functions, therapeutic targeting of DNA-PKcs has regained the momentum as a promising cancer therapy. Reflecting this, numerous highly selective DNA-PKcs inhibitors such as NU7441, M3814, AZD7648, and NU5455 have been developed [15,16,22,23] and are being evaluated in clinical trials (NCT03770689, NCT04172532, NCT04533750). Although some promising preclinical and early-phase clinical results have been reported recently [15,22,24,25,26], the precise management of DNA-PKcs-based therapy for cancer patients remains a challenge due to the physiological role of DNA-PKcs and the lack of biomarkers that predict response to DNA-PKcs inhibitors. Therefore, the identification of biomarkers for DNA-PKcs inhibitor sensitivity is of great importance, not only for the development of precision therapeutic options based on DNA-PKcs inhibitor drugs but also for the mechanistic understanding of DNA-PKcs regulation.

The classical role of DNA-PKcs in DNA damage response has been extensively reviewed elsewhere [3,4,27,28]. Herein, we summarize the recent advances in DNA-PKcs by deliberately focusing on its noncanonical roles in the pathobiology of human cancer, in particular tumor metastasis, metabolism, and immunity. The molecular basis of the pleiotropic role of DNA-PKcs and potential biomarkers that predict therapeutic response to DNA-PKcs inhibitors will also be addressed.

## 2. DNA-PKcs and Cancer

**Figure 1:**
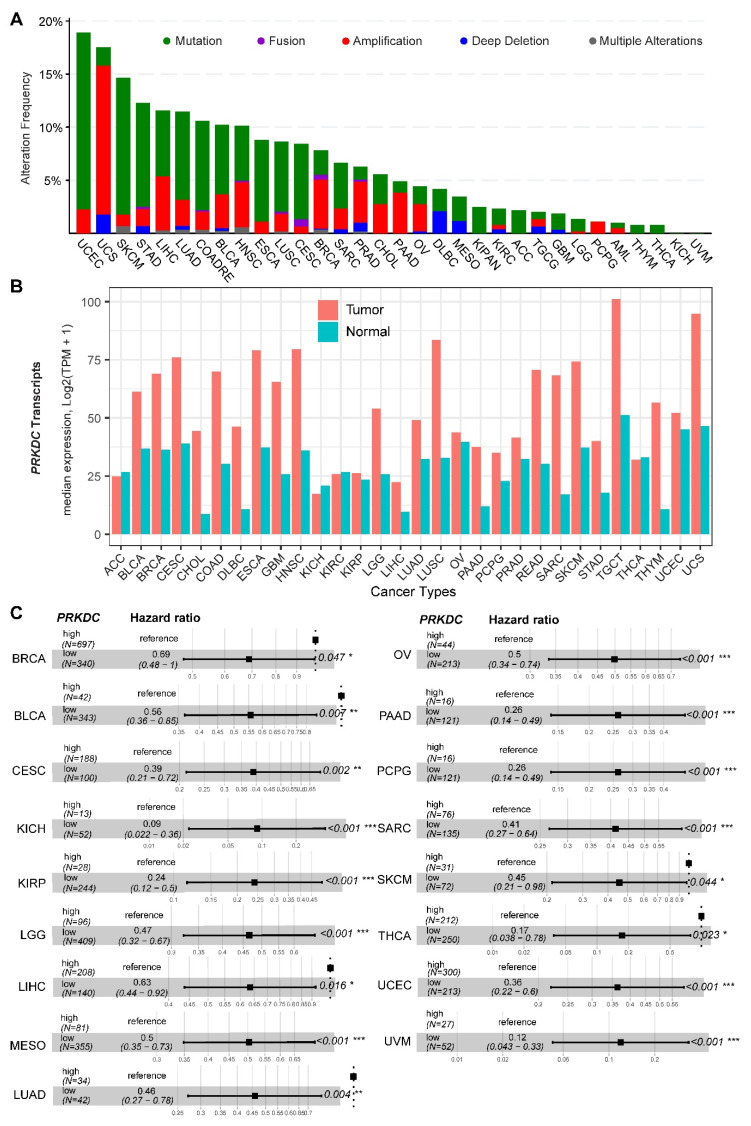
DNA-dependent protein kinase catalytic subunit (DNA-PKcs) in human cancer. (**A**) Genetic landscape of *PRKDC* (encoding DNA-PKcs) across the pan-cancer cohort in The Cancer Genome Atlas (TCGA). Data were downloaded from the cBioPortal database (https://www.cbioportal.org/). *PRKDC* was queried in TCGA pan-cancer cohort. (**B**) Bar plots showing the *PRKDC* gene expression profile across all tumor samples and paired normal tissues. The height of the bar represents the median expression of the indicated tumor (in red) or normal (in blue) tissue. (**C**) Forest blots showing the survival analysis of cancer patients stratified by the gene expression of *PRKDC* across the TCGA pan-solid cancer cohort. Only significant (*p* < 0.05) results were presented. The “high” and “low” expression groups were stratified by the optimal cutoff value using “survminer” and “survival” packages in R software. N, the total number in each group. Scale line indicates the 95% confidence interval for effect estimate for each survival-influencing factor with the hazard ratio showing to the right. ACC, adrenocortical carcinoma; BLCA, bladder urothelial carcinoma; BRCA, breast invasive carcinoma; CESC, cervical squamous cell carcinoma and endocervical adenocarcinoma; CHOL, cholangiocarcinoma; COAD, colon adenocarcinoma; ESCA, esophageal carcinoma; GBM, glioblastoma multiforme; HNSC, head and neck squamous cell carcinoma; KICH, kidney chromophobe; KIRC, kidney renal clear cell carcinoma; KIRP, kidney renal papillary cell carcinoma; LGG, brain lower grade glioma; LIHC, liver hepatocellular carcinoma; LUAD, lung adenocarcinoma; LUSC, lung squamous cell carcinoma; MESO, mesothelioma; OV, ovarian serous cystadenocarcinoma; PAAD, pancreatic adenocarcinoma; PCPG, pheochromocytoma and paraganglioma; PRAD, prostate adenocarcinoma; READ, rectum adenocarcinoma; SARC, sarcoma; SKCM, skin cutaneous melanoma; STAD, stomach adenocarcinoma; TGCT, testicular germ cell tumors; THCA, thyroid carcinoma; UCS, uterine carcinosarcoma; UCEC, uterine corpus endometrial carcinoma; UVM, uveal melanoma. * *p* < 0.05, ** *p* < 0.01, *** *p* < 0.001. The detailed information about the bioinformatic analysis can be found in the Supplementary Material.

**Figure 2:**
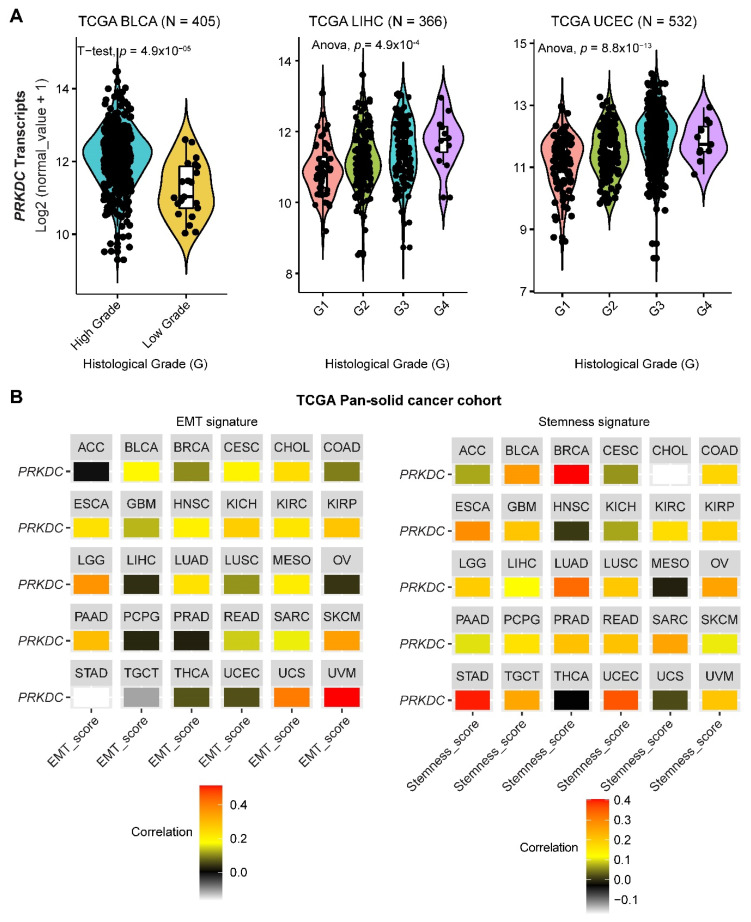
**The role of DNA-PKcs in cancer progression.** (**A**) Violin plots showing the association between *PRKDC* expression and histological grades of TCGA tumors. Note that the information on the histological grades is only available for several cancer types. (**B**) Correlation analysis of the epithelial-to-mesenchymal transition (EMT; left) and stemness (right) signatures and gene expression of *PRKDC* across the TCGA pan-solid cancer cohort. Curated EMT [34,35] and mRNA-based stemness scores [36] derived by the stemness group was used. ACC, adrenocortical carcinoma; BLCA, bladder urothelial carcinoma; BRCA, breast invasive carcinoma; CESC, cervical squamous cell carcinoma and endocervical adenocarcinoma; CHOL, cholangiocarcinoma; COAD, colon adenocarcinoma; ESCA, esophageal carcinoma; GBM, glioblastoma multiforme; HNSC, head and neck squamous cell carcinoma; KICH, kidney chromophobe; KIRC, kidney renal clear cell carcinoma; KIRP, kidney renal papillary cell carcinoma; LGG, brain lower grade glioma; LIHC, liver hepatocellular carcinoma; LUAD, lung adenocarcinoma; LUSC, lung squamous cell carcinoma; MESO, mesothelioma; OV, ovarian serous cystadenocarcinoma; PAAD, pancreatic adenocarcinoma; PCPG, pheochromocytoma and paraganglioma; PRAD, prostate adenocarcinoma; READ, rectum adenocarcinoma; SARC, sarcoma; SKCM, skin cutaneous melanoma; STAD, stomach adenocarcinoma; TGCT, testicular germ cell tumors; THCA, thyroid carcinoma; UCS, uterine carcinosarcoma; UCEC, uterine corpus endometrial carcinoma; UVM, uveal melanoma. The detailed information about the bioinformatic analysis can be found in the Supplementary Material.

DNA-PKcs overexpression is associated with poor prognosis in several cancer cohorts [9,10,11,12,13,14,29]. In the public The Cancer Genome Atlas (TCGA) database, genetic alterations of *PRKDC* (Protein Kinase, DNA-Activated, Catalytic Subunit; encoding DNA-PKcs), e.g., point mutations and copy number amplifications, are common in a variety of cancer types, particularly in uterine corpus endometrial carcinoma (UCEC), uterine carcinosarcoma (UCS), skin cutaneous melanoma (SKCM), stomach adenocarcinoma (STAD), liver hepatocellular carcinoma (LIHC), lung adenocarcinoma (LUAD), and colorectal adenocarcinoma (COADRE) (frequency > 10%) (Figure 1A). High expression of *PRKDC* occurs in the majority of human cancers, compared to the matched normal tissue (Figure 1B), which predicts dismal patient survival in the majority of TCGA cancer types (Figure 1C). However, in hTERT (human telomerase reverse transcriptase)/CDK4 (cyclin-dependent kinase 4) immortalized human bronchial epithelial cells (HBECs), DNA-PKcs knockdown to levels simulating haploinsufficiency dramatically reduced DNA repair capacity with significantly increased transformation efficiency of HBEC lines exposed to bleomycin [8]. The transformation enabled by compromised DNA-PKcs is mainly due to the epigenome reprogramming [8]. Thus, DNA-PKcs may have a dual role, e.g., as a tumor suppressor in premalignant stages but an oncogenic driver in the advanced stage. Supporting this notion, the expression of *PRKDC* is significantly associated with histological grades (Figure 2A).

## 3. DNA-PKcs in Tumor Metastasis and Therapy Resistance

Metastasis and resistance to cancer therapeutics are the main cause of poor clinical outcomes. Intriguingly, there is emerging evidence that DNA-PKcs plays an important role in tumor metastasis and therapy resistance through transcriptional regulation [7,10,20,30,31,32,33].

By analyzing the secretome of isogenic cells differing in DNA-PKcs expression, a previous study [31] showed that, compared to DNA-PKcs-deficient cells, DNA-PKcs-proficient cells secrete higher levels of at least 103 proteins, including 44 metastasis-associated FBLN1 (Fibulin 1), SERPINA3 (Serpin Family A Member 3), MMP-8 (Matrix Metallopeptidase 8), and HSPG2 (Heparan Sulfate Proteoglycan 2), as well as the inhibitors of matrix metalloproteinases, such as α-2M (Alpha-2-macroglobulin) and TIMP-2 (TIMP Metallopeptidase Inhibitor 2) that are associated with a pro-metastatic activity. Consistently, clinical evidence shows that DNA-PKcs expression is dramatically elevated in tumor samples with distant metastasis [7,10,17]. These results suggest that DNA-PKcs may facilitate metastasis by modifying the tumor microenvironment.

The importance of DNA-PKcs for metastasis has been comprehensively demonstrated in prostate cancer, in which DNA-PKcs-mediated gene regulation promotes tumor migration, invasion, and metastases while DNA-PKcs suppression inhibits metastases [7]. In detail, gene set enrichment analysis (GSEA) of the differentially downregulated genes in prostate cancer cells with DNA-PKcs knockdown revealed significant enrichment of genes regulated by MAZ (MYC Associated Zinc Finger Protein), MYC (Myelocytomatosis), and SP1, a well-characterized DNA-PKcs-interacting partner [7]. Of note, DNA-PKcs is highly activated in advanced tumors, which is independent of DNA damage indicators [7]. This study convincingly showed that DNA-PKcs is a potent driver of tumor progression and metastases. Moreover, in an attempt to reveal the underlying mechanisms that mediate the aggressive phenotypes of DNA-PKcs, the same group performed unbiased investigations in multiple tumor models, which revealed that DNA-PKcs is pleiotropic, involved in the regulation of various biological processes beyond DNA repair, such as epithelial–mesenchymal transition (EMT), immune response, and metabolic processes [20,30]. Supporting the findings, a recent study by Kothari et al. showed that, among other kinases, DNA-PKcs is most significantly associated with metastatic progression in high-risk prostate cancer and its inhibition suppressed the growth of both androgen receptor (AR)-dependent and AR-independent prostate cancer cells [30]. Mechanistically, DNA-PKcs interacts with the Wnt transcription factor LEF1 (Lymphoid Enhancer Binding Factor 1) and is critical for LEF1-mediated transcription [30].

By using the previously curated EMT signature [34,35], we found that the gene expression of DNA-PKcs is positively correlated with the EMT signature across a pan-cancer cohort in TCGA (Figure 2B). Interestingly, we also identified a positive correlation between DNA-PKcs and a curated stemness signature [36], defining the status of cancer stemness that plays a crucial role in tumor initiation, relapse, and metastasis in the majority of cancer types (Figure 2B).

In addition to promoting tumor metastasis, preclinical evidence also revealed that DNA-PKcs is associated with primary resistance to chemo- and targeted therapy [32,33,37,38]. However, regarding its role in mediating resistance to therapy, the extent to which these functions of DNA-PKcs are independent of DNA repair has not been well clarified in these studies. Interestingly, a recent study showed that DNA repair-independent functions of DNA-PKcs protect irradiated cells from mitotic slippage and accelerated senescence [18].

## 4. DNA-PKcs in Metabolism

Metabolic dysregulation is a hallmark of cancer [39,40]. Emerging evidence has associated DNA-PKcs with metabolic regulation in physiological and pathological contexts [41,42,43,44]. First, DNA-PKcs can transcriptionally upregulate the genes involved in lipogenesis in response to feeding and insulin signaling [43]. Secondly, DNA-PKcs can facilitate the activation of AMP (adenosine monophosphate)-activated protein kinase (AMPK), a central energy sensor, and, consequently, promote the restoration of energy balance in response to glucose deprivation [42]. Moreover, blocking DNA-PKcs can rescue the age-related metabolic decline [41]. Finally, more recent studies have also revealed that DNA-PKcs regulates cancer metabolism [7,30]. In detail, the c-Myc (cellular Myelocytomatosis) oncoprotein is a master regulator controlling cellular growth and metabolism [45] and DNA-PKcs leads to the activation of the AKT (AKR thymoma)/GSK3 (Glycogen synthase kinase 3) pathway, thereby indirectly stabilizing c-Myc. Indeed, the silencing of *PRKDC* expression decreases Myc protein levels in HeLa cells [46]. Thus, it has been suggested that DNA-PKcs has a dual role. On one side, sufficient DNA-PKcs activity is necessary to maintain genomic integrity. On the other side, dysregulated overexpression of DNA-PKcs may contribute to stabilizing the c-Myc oncoprotein, thereby contributing to the aberrant cellular metabolism characteristic for cancer cells, resulting in oncogenic transformation and aberrant cellular proliferation at the early and late stage of cancer development, respectively.

Autophagy is crucial to maintain cellular metabolic homeostasis and plays a key role in promoting the survival of cancer cells in the case of metabolic stress and facilitating the development of therapeutic resistance [47]. DNA-PKcs-mediated NHEJ functions as the major repair pathway in response to ionizing radiation (IR), providing the rationale for combined treatment with IR and DNA-PKcs inhibitors in various cancers [15,16,48]. However, it remains unclear as to whether the combinatorial effects are exclusively mediated by impaired DSB repair. Accumulating evidence has shown that autophagic cell death also plays a critical role in mediating the effects of IR and DNA-PKcs inhibitor combination [21,48,49,50,51]. Taking advantage of isogenic cancer cell models (human malignant glioma), including M059J cells that lack the DNA-PKcs and the counterpart M059K cells proficient in DNA-PKcs, Daido et al. [48] showed that low-dose IR induced massive autophagic cell death in M059J but not in M059K cells, providing new insights into the molecular mechanisms of combined IR and DNA-PKcs inhibitor treatment, although it is still not clear whether the effect is tissue-specific.

Intriguingly, combined treatment with the DNA-PKcs inhibitor NU7441 and IR showed different effects on non-small cell lung cancer cells (A549, H460, and H1299). Specifically, NU7441 induced G2/M checkpoint and mitotic catastrophe exclusively in H1299, whereas it upregulated autophagy in A549 and induced senescence in H460, suggesting the heterogeneity of DNA-PKcs inhibitor on the choice of cell death. Of note, all the three cell lines harbor *KRAS* (Kirsten Rat Sarcoma Viral Oncogene Homolog) mutations, but differ in amino acid substitutions, which can engage different downstream effectors [52], with the genes co-mutated with *KRAS* also potentially playing a role [52]. Further studies are warranted to identify factors affecting the heterogeneous effects of DNA-PKcs targeted therapy.

Questions remain with regards to how DNA-PKcs interacts with autophagy. A recent study by Puustinen et al. showed that DNA-PKcs acts upstream of AMPK and ULK1 (Unc-51 Like Autophagy Activating Kinase 1) kinase, core regulators of autophagy [21]. Mechanistically, DNA-PKcs interacts with the AMPK complex and phosphorylated its nucleotide-sensing γ1 subunit PRKAG1 /AMPKγ1 (Protein Kinase AMP-Activated Non-Catalytic Subunit Gamma 1) at Ser192 and Thr284. DNA-PKcs-mediated phosphorylation of PRKAG1 inhibited lysosomal localization of the AMPK complex and the starvation-induced association with STK11 (serine/threonine kinase 11). These data suggest that DNA-PKcs-mediated phosphorylation of PRKAG1 primes the AMPK complex to the lysosomal activation by STK11 in cancer cells, thereby linking the function of DNA-PKcs with autophagy and cellular metabolism [21].

## 5. DNA-PKcs in Immunity

NHEJ is required for both V(D)J recombination and class switch recombination (CSR), two Ig (immunoglobulin) gene-diversification processes occurring during B cell development [53], and, consequently, DNA-PKcs inactivation leads to immune deficiency [54,55]. The association of DNA-PKcs with B cell-mediated immunity raises the question of how DNA-PKcs dysregulation is involved in shaping the cancer immune microenvironment.

As a key component of DSB repair and recombination, DNA-PKcs plays a pivotal role in genomic stability. Microsatellite instability (MSI) status has been identified as a biomarker to predict patients who could benefit from immunotherapy. A recent study [56] showed that *PRKDC* mutation was significantly associated with a high mutation load or high-MSI status in cancer on the basis of the TCGA pan-cancer cohort and, more importantly, *PRKDC* knockout and DNA-PKcs inhibitor enhanced the efficacy of immunological checkpoint inhibitors (ICI). These findings highlight *PRKDC* mutation as a potential biomarker for immunotherapy. Moreover, Chen et al. recently investigated the association between *PRKDC* mutations and tumor mutation burden (TMB), tumor microenvironment (TME), and response to ICI by integrated analysis of sequencing data of solid tumors in TCGA (*n* = 4023) and Geneplus (*n* = 3877) [57]. They showed that *PRKDC* mutant tumors have significantly higher TMB than that of *PRKDC* wild-type tumors. Further, solid tumors harboring *PRKDC* mutations were enriched in tumor immunogenicity microenvironments, such as CD8+ T cells, NK (natural killer) cells, and chemokines. Importantly, *PRKDC* mutations were associated with better survival in patients after ICI treatment [57,58]. These results support the use of *PRKDC* mutations as a stratification marker for immunotherapy.

**Figure 3:**
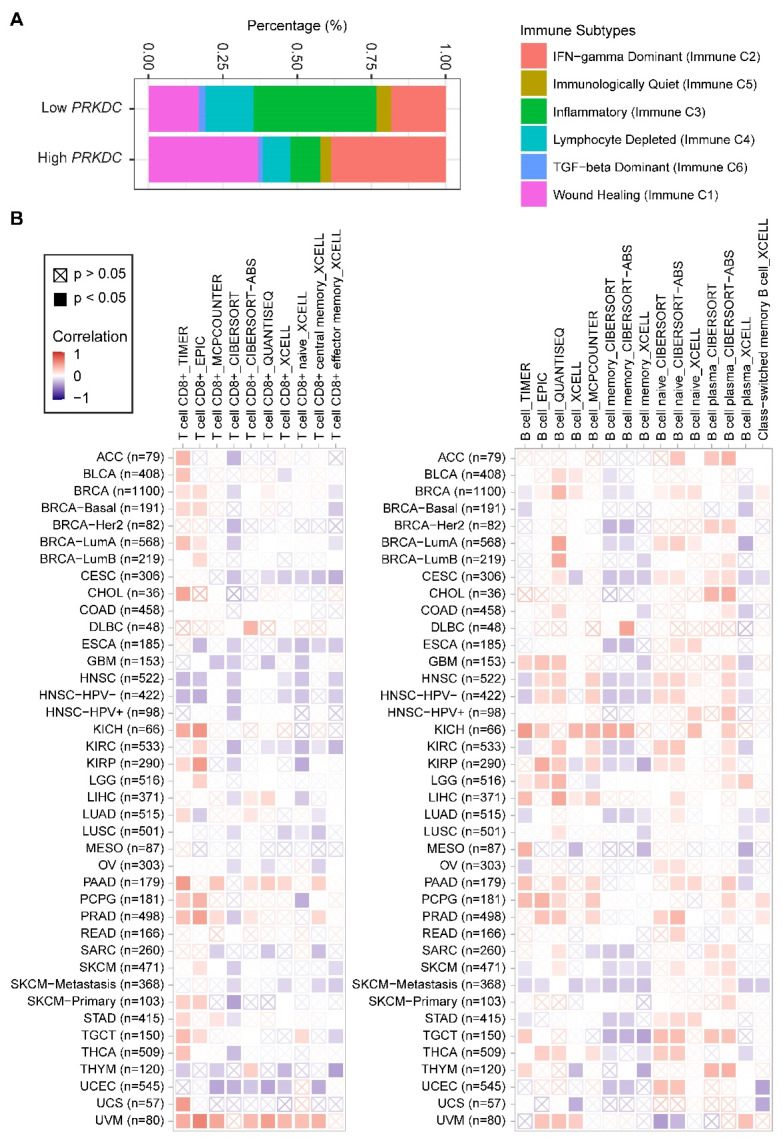
*PRKDC* expression is associated with immune cell infiltrates across diverse cancer types. (**A**) Percentage (*PRKDC* expression low vs. high) of immune subtype models (C1–C6) across TCGA pan-cancer cohort. The genes contained in each signature were evaluated using model-based clustering by p the “mclust” R package. Each sample was finally to be grouped on the basis of its predominance with the C1-C6 signature. The immune subtype models were based on Thorsson et al. (2018) [59]. (**B**) Systematic correlation analysis of immune infiltrates (CD8+, left; CD4+, right) with gene expression of *PRKDC* across TCGA pan-cancer cohort. The number of patients was shown in parenthesis. Data were downloaded from TIMER (version 2.0; with “PRKDC” as a query), a comprehensive resource for systematic analysis of immune infiltrates across diverse cancer types (http://timer.comp-genomics.org/) [60]. The red color indicates a positive correlation (0–1), while the blue color represents a negative correlation (−1–0). The correlation with *p*-value < 0.05 is considered as significant. The correlation coefficient values with *p*-value > 0.05 are marked with a cross. ACC, adrenocortical carcinoma; BLCA, bladder urothelial carcinoma; BRCA, breast invasive carcinoma; CESC, cervical squamous cell carcinoma and endocervical adenocarcinoma; CHOL, cholangiocarcinoma; COAD, colon adenocarcinoma; ESCA, esophageal carcinoma; GBM, glioblastoma multiforme; HNSC, head and neck squamous cell carcinoma; KICH, kidney chromophobe; KIRC, kidney renal clear cell carcinoma; KIRP, kidney renal papillary cell carcinoma; LGG, brain lower grade glioma; LIHC, liver hepatocellular carcinoma; LUAD, lung adenocarcinoma; LUSC, lung squamous cell carcinoma; MESO, mesothelioma; OV, ovarian serous cystadenocarcinoma; PAAD, pancreatic adenocarcinoma; PCPG, pheochromocytoma and paraganglioma; PRAD, prostate adenocarcinoma; READ, rectum adenocarcinoma; SARC, sarcoma; SKCM, skin cutaneous melanoma; STAD, stomach adenocarcinoma; TGCT, testicular germ cell tumors; THCA, thyroid carcinoma; UCS, uterine carcinosarcoma; UCEC, uterine corpus endometrial carcinoma; UVM, uveal melanoma. The detailed information about the bioinformatic analysis can be found in the Supplementary Material.

Nevertheless, how the expression level of DNA-PKcs affects the tumor immune microenvironment is still unknown. Our analysis of a pan-cancer cohort in the TCGA showed that solid tumors expressing high levels of *PRKDC* differ in the immune subtype from those with low *PRKDC* expression (Figure 3A), on the basis of previously curated immune subtype models [59]. Specifically, high *PRKDC* tumors were enriched with IFN (interferon)-γ-dominant and wound healing subtypes, and *PRKDC* expression significantly correlates CD8+ T cell and B cell signatures across different cancer types (Figure 3B) [60]. Despite the potential roles of DNA-PKcs in cancer immunity, it remains unclear as to whether targeting DNA-PKcs could affect the efficacy of ICI treatment. To improve the immunotherapy for melanoma patients, Tsai and colleagues used the unbiased high-throughput flow cytometry-based screening to identify and characterize candidate therapies that might synergize with and augment T-cell immunotherapy efficacy [19]. Surprisingly, NU7441, a selective inhibitor of DNA-PKcs [23], was identified as one of the two lead therapies, as NU7441 could alter a variety of immunomodulatory proteins, e.g., CD55, CD73, CD155, programmed death-ligand 1 (PD-L1), nerve growth factor receptor (NGFR), and HLA (human leukocyte antigen) class I in a heterogeneous panel of melanomas, leading to proliferative inhibition of melanoma cells [19]. Consequently, targeting DNA-PKcs enhances the efficacy of immunotherapies. These findings provided mechanistic insights and a strong rationale for co-targeting DNA-PKcs to improve immunotherapy.

## 6. DNA-PKcs Interactors

Although the pleiotropic role of DNA-PKcs—DNA repair-dependent or -independent—have been well established, the way in which the activity of DNA-PKcs is mediated is still not fully elucidated. DNA-PKcs and the regulatory subunits Ku70/80 form with a 1:1:1 stoichiometry the DNA-PK complex, which has serine/threonine protein kinase activity and plays a pivotal role in the repair and recovery from DNA damage [18,61]. In addition, DNA-PKcs phosphorylates a variety of transcription factors and the large subunit of RNA polymerase II, thereby indirectly regulating gene transcription [2,61]. Additionally, DNA-PKcs is a large protein with multiple domains that can potentially interact with plenty of other proteins within a cell [42,62,63,64,65], which might contribute to the multifaceted roles of DNA-PKcs [20].

A recent study by Song and colleagues systematically explored the DNA-PKcs–RNA interactome and characterized the genome-wide landscape of DNA-PKcs-associated RNAs on the basis of RNA immunoprecipitation coupled with next-generation sequencing (RIP-seq) [62]. Approximately 500 RNAs were co-precipitated with DNA-PKcs with a stringent cutoff. Subsequent pathway enrichment analysis showed that the RNAs bound to DNA-PKcs were mainly involved in the focal adhesion and receptor–ECM (extracellular matrix) interaction signaling pathways, indicating that DNA-PKcs may control a variety of biological processes partially through its RNA-binding activity.

**Figure 4:**
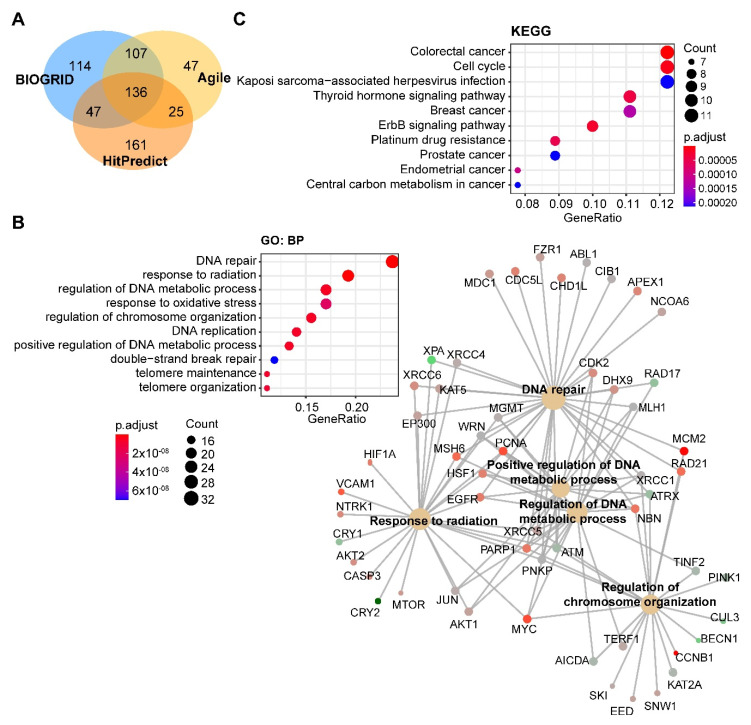
**Pathway analysis of DNA-PKcs interacting proteins.** (**A**) Venn diagram showing the proteins commonly interacting with DNA-PKcs on the basis of three publicly curated datasets (Agile Protein Interactomes DataServer (http://cicblade.dep.usal.es:8080/APID/init.action), BioGRID (version 4.0; https://thebiogrid.org/), and HitPredict (http://www.hitpredict.org/)) using *PRKDC* as input. Venn diagram was generated using “VennDiagram” package in R software. (**B**,**C**) Top 10 significantly enriched GO (Gene Ontology; biological process, BP) and Kyoto Encyclopedia of Genes and Genomes (KEGG) (**C**) pathways based on proteins commonly interacting with DNA-PKcs. Cnetplot in B (right) listed gene names of the proteins enriched in the GO pathway. The KEGG and GO pathway enrichment analyses were performed using the R package “clusterprofiler”. The detailed information about the bioinformatic analysis can be found in the Supplementary Material.

Through integrated analysis of the publicly curated protein interaction databases (Protein—interacting data downloaded from Agile Protein Interactomes DataServer (APID, http://cicblade.dep.usal.es:8080/APID/init.action); BioGRID, version 4.0; https://thebiogrid.org/; HitPredict, http://www.hitpredict.org/), we identified 136 common interacting proteins with DNA-PKcs (Figure 4A). Pathway analysis showed that these proteins are enriched in diverse cellular processes apart from DNA repair (Figure 4B), e.g., regulation of processes related to DNA metabolism, response to oxidative stress, and telomere maintenance, which were also observed in previous studies [44,65,66]. Kyoto Encyclopedia of Genes and Genomes (KEGG) pathway analysis showed that these proteins were significantly enriched in multiple cancer types (Figure 4C).

Overall, the evidence above suggests that DNA-PKcs can regulate a variety of pathways while its own activity is modulated by a network of interacting pathways. Thus, DNA-PKcs serves as a central node of multiple interconnected signaling pathways to orchestrate diverse cellular processes including DNA repair, metabolism, and transcription.

## 7. Biomarkers for Sensitivity to DNA-PKcs Targeted Therapy

DNA-PKcs inhibitors have been shown to be essential in several cancers and these inhibitors alone have been shown to strongly affect the survival of cancer cells of different lineages [67,68,69] despite ambivalent responses [50,67], highlighting the need for biomarkers to improve the clinical utility of DNA-PKcs-targeted therapy.

*TP53* (Tumor Protein P53) mutational status was identified as a biomarker for therapeutic response to combined treatment with radiation and DNA-PKcs targeted therapy [70]. Specifically, cancer cells with dysfunctional p53 were unable to fully arrest their cell cycle and thus entered S and M phases with unrepaired DNA, leading to mitotic catastrophe and apoptotic cell death. In contrast, in cancer cells with wild-type *TP53*; ataxia telangiectasia-mutated (*ATM*); and its targets, p53 and checkpoint kinase 2 (CHK2), were more strongly activated by the addition of DNA-PKcs inhibitor M3814, leading to a complete p53-dependent cell-cycle block and premature cell senescence [70]. Supporting this notion, pharmacological or genetic abrogation of DNA-PKcs leads to apoptotic death of ATM-defective cells, highlighting DNA-PKcs inhibitors being a promising treatment for ATM-defective malignancies. By integrating the mutational landscape of 1319 cancer-associated genes with sensitivity data of DNA-PKcs inhibition across 67 cell lines [71], Dietlein and colleagues identified a considerable number of genes involved in homologous recombination-mediated DNA repair, including *BRCA1* (Breast And Ovarian Cancer Susceptibility Protein 1)*, BRCA2* (Breast And Ovarian Cancer Susceptibility Protein 2)*, ATM* (Ataxia Telangiectasia Mutated)*, PAXIP1* (paired box paired box 1), and *RAD50* (DNA repair protein RAD50), whose mutations led to non-oncogene addiction to DNA-PKcs. In particular, mutations in the mismatch repair gene *MSH3* are the most significant predictors of DNA-PKcs addiction. Consequently, DNA-PKcs inhibition robustly induced apoptosis in *MSH3*-mutant tumors in vitro and in vivo. Thus, the mutational status in *TP53, ATM,* and *MSH3* should be taken into account for the future design of clinical trials.

Synergistic combination therapy with DNA-PKcs inhibitors has also been investigated, especially with chemo- and radiotherapy [22,72,73,74]. More recently, combined PARP (Poly (ADP-ribose) polymerase)-targeted therapy and DNA-PKcs inhibitor have been demonstrated to synergistically inhibit tumor growth in both orthotopic mouse and patient-derived xenograft models of hepatocellular carcinoma [24]. Importantly, the combination regimen showed low toxicity, as no significant influence on liver, kidney, heart, and body weight was observed [24].

It has been shown that DNA-PKcs is important for sustained activation of the AKT signaling pathway [6,33,75,76,77], which is interlinked with EMT, therapy resistance, and cancer metabolism [78,79]. These observations suggest that activation of the AKT signaling pathway in tumors may serve as a potential biomarker for response to DNA-PKcs-targeted therapy.

**Figure 5:**
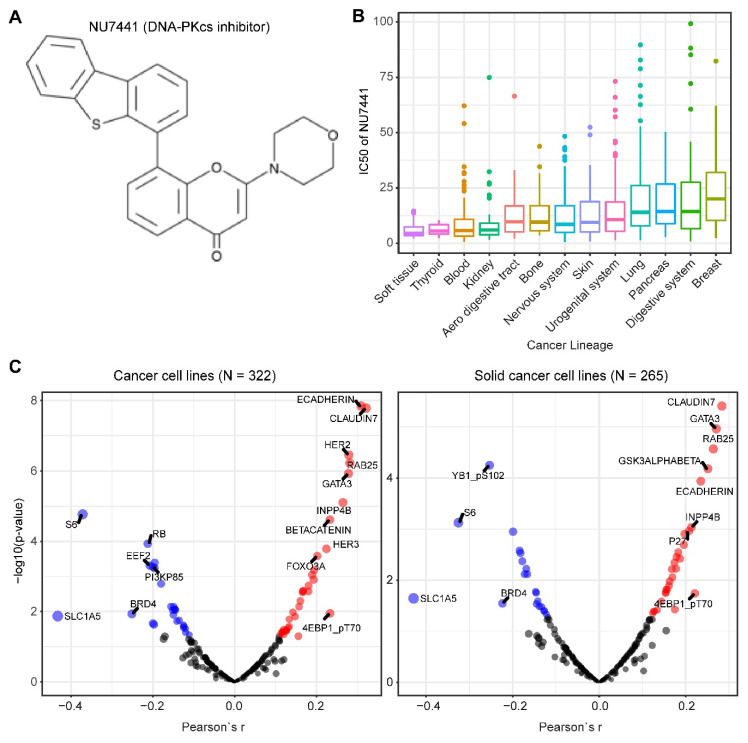
**Biomarkers predicting therapeutic response to the DNA-PKcs inhibitor NU7441.** (**A**) The molecular structure of NU7441 compound. (**B**) IC_50_ (half maximal inhibitory concentration) values of cancer cell lines with different lineages to NU7441. Data were downloaded and reanalyzed from GDSC (Genomics of Drug Sensitivity in Cancer, https://www.cancerrxgene.org/). (**C**) Integrated correlation analysis of IC_50_ of NU7441 and protein array data across cancer cell lines (left) and solid cancer cell lines (right). Red dots indicate proteins whose expression level significantly (*p* < 0.05) positively correlates with the effectiveness (IC_50_ value) of NU7441, while the blue dots represent the significantly negatively correlated proteins. The protein array data were downloaded from TCPA (The Cancer Proteome Atlas) cancer cell line cohort (https://tcpaportal.org/), which contains 216 proteins critical for cancer. The detailed information about the bioinformatic analysis can be found in the Supplementary Material.

We mined drug sensitivity data of NU7441, a selective DNA-PKcs inhibitor [23], in GDSC (Genomics of Drug Sensitivity in Cancer, https://www.cancerrxgene.org/) and showed that cancer cell lines from soft tissue and thyroid are particularly sensitive to NU7441, whereas cancer cell lines with other lineages display highly heterogeneous responses to NU7441 (Figure 5A,B). To systematically probe the biomarkers for response to DNA-PKcs inhibitors, we correlated the drug response profiles of NU7441 and the protein reverse phase (RPPA) dataset of TCPA (The Cancer Proteome Atlas) cancer cell line cohort (https://tcpaportal.org/), which identified several proteins whose expression is significantly correlated, negatively and positively, with the IC_50_ value of NU7441 (Figure 5C). It is noteworthy that a negative correlation indicates that a higher protein content correlates with a lower IC_50_ value and thus represents a biomarker of sensitivity to treatment and vice versa. S6, a key downstream effector of the AKT/mTOR (mechanistic target of rapamycin) signaling pathway, is most negatively correlated with NU7441 (high S6 level and low IC_50_), which is in line with the role of DNA-PKcs in activating AKT (Figure 5C) [6,33,75,76,77]. Of particular interest, E-cadherin and claudin 7 (typical epithelial markers) are positively correlated with the IC_50_ value (Figure 5C), indicating that DNA-PKcs inhibitors preferentially target mesenchymal cells that are actively involved in tumor metastasis and therapy resistance [34,80].

Of note, although numerous clinical trials (NCT03724890, NCT03770689, NCT03983824, NCT04071236, NCT04172532, NCT02316197, NCT04092270) with DNA-PKcs inhibitors are ongoing, few involve biomarker-guided patient stratification, which is critical, as we discuss above.

## 8. Toxicity Concern of DNA-PKcs Targeted Therapy

DNA-PKcs plays essential functions in both normal tissues and tumors [3,4,5,20,28,44], raising the possibility of toxicity concern of DNA-PKcs-targeted therapy in vivo. Mice with *PRKDC* knockout exhibit severe combined immune-deficiency and increased sensitivity to ionizing radiation [81,82]. Further, compared to wild-type mice, DNA-PKcs-null mice have reduced viability and display an earlier onset of aging-related pathologies, e.g., thymus lymphoma, infection, and intestinal atrophy, as well as compromised telomere activity due to the role of DNA-PKcs in the maintenance of the telomere length [83]. Notably, the toxicity of DNA-PKcs inhibitors is expected to be significantly increased when administered in combination with DNA-damaging agents, given that they directly block the DNA repair machinery. Note that DNA-PKcs deletion is not identical as pharmacological inhibition [82], with the latter typically inducing a transient inhibition of its kinase activity that may explain the reported tolerability of selective DNA-PKcs inhibitors [7,15,16], although long-term toxicity data are still missing.

The potential side effects may be overridden by co-targeting other genes, e.g., the aforementioned *MSH3, ATM, BRCA1*, and *BRCA2*, whose inactivation is synthetic lethal with DNA-PKcs inhibitors.

The tumor hypoxic microenvironment plays a critical role in malignant phenotypes and therapy resistance. The importance of DNA-PKcs for tumors cells in response to hypoxic microenvironment has been well documented [84,85,86], which can also be taken into account to enhance the efficacy of DNA-PKcs inhibitors [87,88], thereby preferentially affecting the tumor cells and reducing the adverse effects. Intriguingly, Wong et al. recently showed that SN38023, a novel prodrug, can be metabolized to a potent DNA-PKcs inhibitor (IC87361) selectively in hypoxic cells [88], such that SN38023 selectively inhibited radiation-activated Ser2056 autophosphorylation of DNA-PKcs and radio-sensitized cancer cells under anoxia [88], indicating the promise to exploit hypoxia for selective delivery of DNA-PKcs inhibitors to target tumor cells.

## 9. Conclusions

Although DNA-PKcs in DNA repair has been well studied, emerging evidence suggests that DNA-PKcs also regulates several other cancer-related cellular processes such as tumor metastasis and resistance to anticancer therapy, metabolism, and immunity. A key question is how the multifaceted roles of DNA-PKcs are achieved. Recent progress and our analysis of public datasets have provided insightful clues for these questions, e.g., the multiple intracellular interactors of DNA-PKcs may underlie its multifaceted roles in cancer. However, several other questions, e.g., how DNA-PKcs is regulated differently in cancer than in normal tissue, what are the optimal biomarkers for response to DNA-PKcs-targeted therapy, and how to minimize the potential toxicity of DNA-PKcs inhibitors, are still largely unanswered. The evidence provided by this review shows that sensitivity to DNA-PKcs inhibitors is correlated with the activity of several signaling pathways, in particular the PI3K (Phosphoinositide 3-kinase)/AKT/mTOR pathway and epithelial/mesenchymal state in cancer. Further elucidation of these questions with genome-wide genetic and/or pharmacological studies (e.g., DNA-PKcs inhibitor-based shRNA [short hairpin RNA]/CRISPR [clustered regularly interspaced short palindromic repeats] screens, integrated pharmaco-transcriptomic analyses) will deepen our knowledge about the molecular mechanisms determining the therapeutic responses to DNA-PKcs inhibitors [89,90], and will contribute greatly to the future design of precision treatment of cancer patients with DNA-PKcs-targeted therapy.

## Supplementary Materials

The following are available online at https://www.mdpi.com/2072-6694/12/11/3389/s1. Methods: bioinformatic analysis and public databases.
