# Supplementary material for: Beyond DNA Repair: DNA-PKcs in Tumor Metastasis, Metabolism and Immunity
Source: Cancers (Basel). 2020 Nov 16;12(11):3389. doi: 10.3390/cancers12113389 (PMC7698146; doi:10.3390/cancers12113389)
Supplement: Supplementary file 1 [file cancers-12-03389-s001.pdf]

cancers

MDPI

Beyond DNA Repair: DNA-PKcs in Tumor Metastasis, Metabolism and Immunity

Haitang Yang [1], Feng Yao [1], Thomas M. Marti [2,3], Ralph A. Schmid [2,3,*] and Ren-Wang Peng [2,3,*]

Methods

Public databases and bioinformatic analysis

1.1. TCGA and TCPA datasets

Transcriptomic and whole-genome exome sequencing data of TCGA (The Cancer Genome Atlas Program) pan-cancer cohort were downloaded from cBioPortal (https://www.cbioportal.org/), and only primary tumor samples and the matched normal tissue were included. Normalized level 3 data of reverse phase protein array (RPPA) were downloaded from The Cancer Proteome Atlas (TCPA) database (https://tcpaportal.org/tcpa/) [1], which quantified 216 proteins of cancer cell lines based on the Broad Institute Cancer Cell Line Encyclopedia (CCLE) project (https://portals.broadinstitute.org/ccle). Here, TCPA Level 3 data were utilized, and the proteomic data normalization was processed as follows (For the details please refer to the FAQ section: https://tcpaportal.org/mclp/#): 1) Calculate the median for each protein across all the samples. 2) Subtract the median (from step 1) from values within each protein. 3) Calculate the median for each sample across all proteins. 4) Subtract the median (from step 3) from values within each sample.

1.2. Drug response profile of NU7441

The drug sensitivity data of cancer cell lines in response to NU7441, a selective DNA-PKcs inhibitor, were downloaded from GDSC (Genomics of Drug Sensitivity in Cancer) database (https://www.cancerrxgene.org/). The sensitivity data were reflected using IC50 (half maximal inhibitory concentration) values. Integrated correlation analysis of IC50 of NU7441 and protein array data (from TCPA) across solid cancer cell lines was performed to reveal the proteins whose expression correlates with the IC50 value of NU7441.

1.3. DNA-PKcs interactors

Protein-interacting data querying for PRKDC were downloaded from Agile Protein Interactomes DataServer (http://cicblade.dep.usal.es:8080/APID/init.action), BioGRID (version 4.0; https://thebiogrid.org/), and HitPredict (http://www.hitpredict.org/). Venn diagram was generated using “VennDiagram” package in R software to display the intersection among these three datasets. The proteins commonly interacting with DNA-PKcs were selected to explore their biological functions, such as Gene Ontology (GO), Kyoto Encyclopedia of Genes and Genomes (KEGG), using the R package “clusterprofiler” [2]. Cnetplot was used to list gene names of the proteins enriched in the GO pathway.

1.4. Tumor immune infiltrates

Tumor-infiltrating immune cell profiles across the TCGA pan-cancer cohort were downloaded from TIMER (version 2.0), a comprehensive resource for systematic analysis of immune infiltrates across diverse cancer types (http://timer.comp-genomics.org/) [3]. Curated immune subtype models (C1-C6) were based on a previous study [4]. The genes contained in each signature were evaluated using model-based clustering by p the “mclust” R package. Each sample was finally to be grouped based on its predominance with the C1-C6 signature.

1.5. EMT and stemness signature score

Curated EMT (epithelial-mesenchymal transition) scored as the sum of a mesenchymal gene set (FN1 + VIM + ZEB1 + ZEB2 + TWIST1 + TWIST2 + SNAI1 + SNAI2 + CDH2) minus that of epithelial genes (CLDN4 + CLDN7 + TJP3 + MUC1 + CDH1) [5,6]. The curated mRNA-based Stemness Scores [7] derived by the Stemness group was used and downloaded from UCSC Xena [8].

1.6. Survival Analysis

Forest blots showing the survival analysis of cancer patients stratified by the gene expression of PRKDC across the TCGA pan-solid cancer cohort. The "high" and "low" expression groups were stratified by the optimal cutoff value using "survminer" and "survival" packages in R software (version 3.6.3) [9-11]. $p < 0.05$ was considered statistically significant.

Reference

1. Li, J.; Lu, Y.; Akbani, R.; Ju, Z.; Roebuck, P.L.; Liu, W.; Yang, J.Y.; Broom, B.M.; Verhaak, R.G.; Kane, D.W., et al. TCPA: a resource for cancer functional proteomics data. *Nat Methods* **2013**, *10*, 1046-1047, doi:10.1038/nmeth.2650.
2. Yu, G.; Wang, L.G.; Han, Y.; He, Q.Y. clusterProfiler: an R package for comparing biological themes among gene clusters. *OMICS* **2012**, *16*, 284-287, doi:10.1089/omi.2011.0118.
3. Li, T.; Fu, J.; Zeng, Z.; Cohen, D.; Li, J.; Chen, Q.; Li, B.; Liu, X.S. TIMER2.0 for analysis of tumor-infiltrating immune cells. *Nucleic Acids Res* **2020**, *48*, W509-W514, doi:10.1093/nar/gkaa407.
4. Thorsson, V.; Gibbs, D.L.; Brown, S.D.; Wolf, D.; Bortone, D.S.; Ou Yang, T.H.; Porta-Pardo, E.; Gao, G.F.; Plaisier, C.L.; Eddy, J.A., et al. The Immune Landscape of Cancer. *Immunity* **2018**, *48*, 812-830 e814, doi:10.1016/j.immuni.2018.03.023.
5. Yang, H.; Liang, S.Q.; Xu, D.; Yang, Z.; Marti, T.M.; Gao, Y.; Kocher, G.J.; Zhao, H.; Schmid, R.A.; Peng, R.W. HSP90/AXL/eIF4E-regulated unfolded protein response as an acquired vulnerability in drug-resistant KRAS-mutant lung cancer. *Oncogenesis* **2019**, *8*, 45, doi:10.1038/s41389-019-0158-7.
6. Kitai, H.; Ebi, H.; Tomida, S.; Floros, K.V.; Kotani, H.; Adachi, Y.; Oizumi, S.; Nishimura, M.; Faber, A.C.; Yano, S. Epithelial-to-Mesenchymal Transition Defines Feedback Activation of Receptor Tyrosine Kinase Signaling Induced by MEK Inhibition in KRAS-Mutant Lung Cancer. *Cancer Discov* **2016**, *6*, 754-769, doi:10.1158/2159-8290.CD-15-1377.
7. Malta, T.M.; Sokolov, A.; Gentles, A.J.; Burzykowski, T.; Poisson, L.; Weinstein, J.N.; Kaminska, B.; Huelsken, J.; Omberg, L.; Gevaert, O., et al. Machine Learning Identifies Stemness Features Associated with Oncogenic Dedifferentiation. *Cell* **2018**, *173*, 338-+, doi:10.1016/j.cell.2018.03.034.
8. Goldman, M.J.; Craft, B.; Hastie, M.; Repecka, K.; McDade, F.; Kamath, A.; Banerjee, A.; Luo, Y.; Rogers, D.; Brooks, A.N., et al. Visualizing and interpreting cancer genomics data via the Xena platform. *Nat Biotechnol* **2020**, *38*, 675-678, doi:10.1038/s41587-020-0546-8.
9. Yang, H.; Xu, D.; Yang, Z.; Yao, F.; Zhao, H.; Schmid, R.A.; Peng, R.W. Systematic Analysis of Aberrant Biochemical Networks and Potential Drug Vulnerabilities Induced by Tumor Suppressor Loss in Malignant Pleural Mesothelioma. *Cancers (Basel)* **2020**, *12*, doi:10.3390/cancers12082310.
10. Yang, H.; Xu, D.; Gao, Y.; Schmid, R.A.; Peng, R.W. The Association of BAP1 Loss-of-Function With the Defect in Homologous Recombination Repair and Sensitivity to PARP-Targeted Therapy. *J Thorac Oncol* **2020**, *15*, e88-e90, doi:10.1016/j.jtho.2020.02.028.
11. Xu, D.; Yang, H.; Yang, Z.; Berezowska, S.; Gao, Y.; Liang, S.Q.; Marti, T.M.; Hall, S.R.R.; Dorn, P.; Kocher, G.J., et al. Endoplasmic Reticulum Stress Signaling as a Therapeutic Target in Malignant Pleural Mesothelioma. *Cancers (Basel)* **2019**, *11*, doi:10.3390/cancers11101502.

© 2020 by the authors. Submitted for possible open access publication under the terms and conditions of the Creative Commons Attribution (CC BY) license (http://creativecommons.org/licenses/by/4.0/).
